# Clinical Association of Negative Lymph Nodes With Adjuvant Chemotherapy in Patients With T3N0 Rectal Cancer

**DOI:** 10.1155/grp/3241615

**Published:** 2025-02-18

**Authors:** Dongxu Lei, Zhanzhen Liu, Xinyi Kang, Ziwei Zeng, Hao Xie, Tanxing Cai, Fujin Ye, Li Xiong, Wenxin Li, Zhenxing Liang, Xiaobin Zheng, Shuangling Luo, Huashan Liu

**Affiliations:** ^1^Department of General Surgery (Colorectal Surgery), The Sixth Affiliated Hospital, Sun Yat-sen University, Guangzhou, Guangdong, China; ^2^Guangdong Provincial Key Laboratory of Colorectal and Pelvic Floor Diseases, The Sixth Affiliated Hospital, Sun Yat-sen University, Guangzhou, Guangdong, China; ^3^Biomedical Innovation Center, The Sixth Affiliated Hospital, Sun Yat-sen University, Guangzhou, Guangdong, China; ^4^Zhongshan Medicine School, Sun Yat-sen University, Guangzhou, Guangdong Province, China; ^5^Guangdong Provincial Key Laboratory of Digestive Cancer Research, The Seventh Affiliated Hospital of Sun Yat-sen University, Shenzhen, Guangdong, China

**Keywords:** adjuvant chemotherapy, negative lymph nodes, prognosis, rectal cancer

## Abstract

**Background:** The use of adjuvant chemotherapy in patients with stage T3N0 rectal cancer following total mesorectal excision (TME) is debated. This study is aimed at investigating the clinical significance of negative lymph node (NLN) counts in patients with T3N0 rectal cancer, particularly in relation to adjuvant chemotherapy.

**Methods:** This retrospective analysis examined 311 patients with T3N0 rectal cancer who underwent radical resection at the Sixth Affiliated Hospital of Sun Yat-sen University between August 2014 and December 2021. The optimal cutoff for NLN counts was determined using receiver operating characteristic (ROC) curves. Clinicopathological characteristics and clinical outcomes were compared between the high and low NLN groups. Overall survival (OS) and disease-free survival (DFS) were used to evaluate the efficacy of adjuvant chemotherapy.

**Results:** The optimal cutoff for NLNs was 21. Of the 311 patients, 141 were categorized into the high NLN group and 170 into the low NLN group. Patients with NLNs ≥ 21 had significantly better 5-year OS (99.3% vs. 88.2%, *p* < 0.05) and 5-year DFS (92.2% vs. 79.4%, *p* < 0.05) compared to those with low NLNs. Multivariate Cox analysis revealed that NLN count was an independent prognostic factor for OS (hazard ratio (HR) = 0.078, 95% confidence interval (CI): 0.011–0.582, *p* = 0.013) and DFS (HR = 0.417, 95% CI: 0.213–0.815, *p* = 0.011). Subgroup analysis indicated that adjuvant chemotherapy significantly improved OS (*p* < 0.05) and DFS (*p* < 0.05) in the low NLN group.

**Conclusion:** NLN count is an independent prognostic factor in patients with T3N0 rectal cancer. Patients with low NLN counts (NLN < 21) may benefit from adjuvant chemotherapy.

## 1. Introduction

Total mesorectal excision (TME) has significantly improved surgical outcomes for patients with rectal cancer [1, 2]. Despite this, approximately 30% of patients who undergo TME will experience disease recurrence [2–4]. Adjuvant chemotherapy (AC) is commonly administered after surgery to prevent disease recurrence [5, 6]. However, the advantages of AC in patients with T3N0 rectal cancer remain controversial [7]. The National Comprehensive Cancer Network (NCCN) guidelines recommend AC for T3N0 rectal cancer [8], while studies such as EORTC 22921, Dutch Colorectal Cancer Group (DCCG), and I-CNR-RT have reported no significant benefit of AC for this patient group [9–11]. The existing research highlights the need to identify individuals who could benefit from AC in this patient population.

With the application of neoadjuvant chemoradiotherapy (nCRT) and TME surgery, some rectal cancer patients achieved pathologic complete response (pCR) [12]. Although pCR is associated with a favorable prognosis, the need for AC in pCR patients remains controversial. A meta-analysis indicates that AC following pCR significantly improves overall survival (OS) and 5-year OS rates [13]. Additionally, Polanco et al. have shown that AC can enhance 5-year OS in pCR patients [14]. The NCCN guidelines also recommend AC for these patients [15]. However, other studies suggest that for certain pCR patients omitting AC may be safe [16–18]. Therefore, identifying patients who are likely to benefit from AC is critical for optimizing treatment strategies.

The number of negative lymph nodes (NLNs) has been found to be associated with the immune status of the host, and low NLNs are typically indicative of poor prognosis [19]. Studies in breast cancer by San et al. reported a 5-year disease-free survival (DFS) of 69.5% for patients with low NLNs and 87.5% for those with high NLNs [20]. Similarly, Hsu et al. demonstrated a 5-year survival rate of only 26.4% for esophageal cancer patients with low NLNs [21]. In the case of rectal cancer, Sun et al. found that low NLNs were associated with worse outcomes [22]. Building on these findings, we hypothesized that T3N0 rectal cancer patients with low NLNs may benefit from AC. To test this hypothesis, we conducted a retrospective study to assess the effects of NLNs on AC in patients with T3N0 rectal cancer. Our study contributes to clinical decision-making regarding the use of AC in patients with T3N0 rectal cancer.

## 2. Methods

### 2.1. Study Design

Ethical approval for this study was obtained from the Research Ethics Committee at the Sixth Affiliated Hospital of Sun Yat-sen University, and the study adhered to the guidelines set forth in the Declaration of Helsinki (Project Number: 2023ZSLYEC-125). As the data used in the study were anonymized, informed consent was waived.

The study enrolled patients with T3N0 rectal cancer who had undergone surgery at the Sixth Affiliated Hospital of Sun Yat-sen University between August 2014 and December 2021. The clinical and pathological data for all patients were obtained from the Rectal Cancer Database at the Sixth Affiliated Hospital of Sun Yat-sen University. Any missing information was further retrieved and supplemented through the electronic medical record system. Inclusion criteria were as follows: (1) pathologically confirmed rectal cancer; (2) patients underwent radical resection; and (3) postoperative pathology was confirmed as T3N0 stage.

Exclusion criteria included: (1) previous or simultaneous malignancy; (2) emergency or palliative surgical resection; (3) preoperative neoadjuvant chemoradiotherapy; (4) treatment with local excision or wait-and-watch strategy; (5) death within 1 month after surgery; and (6) incomplete clinical information.

Patient characteristics including gender, age, pathological stage, histological classification, nerve infiltration and vascular invasion, AC status, and survival status (survived, deceased, or time to disease progression) were collected through the electronic medical record system and follow-up system.

### 2.2. Pathologic Examination and Follow-Up

The lymph nodes (LNs) were identified and separated from the resected specimens by at least two experienced pathologists according to standard protocols. In cases where fewer than 12 LNs were retrieved, they were reexamined to ensure accurate staging of rectal cancer.

To monitor patients' health status, follow-ups were conducted within 1 year of surgery every 3 months, every 6 months between 1 and 2 years after surgery, and every 12 months thereafter (i.e., postoperative follow-ups at 3, 6, 9, 12, 18, 24, 36, 48, and 60 months). The primary outcomes of this study were OS and DFS. DFS was defined as the time from surgery to local or distant failure or death, while OS was defined as the time from diagnosis to death from any cause. This study followed up until the date of death or end of the study's follow-up (October 2022).

### 2.3. Statistical Analysis

The optimal cutoff for NLN counts was determined using receiver operating characteristic (ROC) curves. Categorical characteristics were compared using chi-square test or Fisher's exact test, while continuous variables were described as means ± standard deviation or median with interquartile range (IQR) and analyzed using analysis of variance or the appropriate Kruskal–Wallis test. Survival curves were analyzed using the Kaplan–Meier method, and differences were compared using the log-rank test. Risk factors for OS and DFS outcomes were analyzed using Cox regression models. Variables that were significant in the univariate Cox-proportional hazard model were included in the multivariate Cox-proportional hazard model. SPSS for Windows (Version 27.0, IBM) was used for all statistical analyses. All *p* values were two-sided, and *p* < 0.05 was considered statistically significant.

## 3. Results

### 3.1. Patient Characteristics

**Figure 1:**
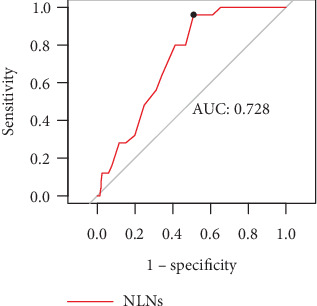
Cutoff points for NLNs determined by ROC. Cutoff points for NLNs determined by ROC, area under the ROC curve: 0.73. The sensitivity and specificity of the point with highest accuracy were 96.0% and 49.0%.

**Figure 2:**
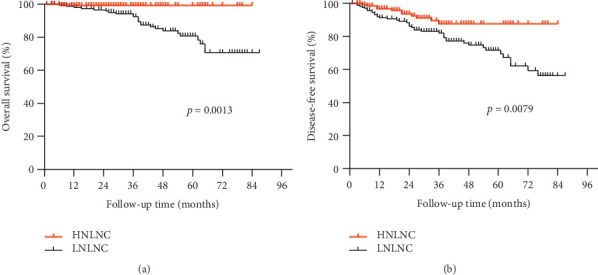
Survival outcomes according to NLNs in all T3N0 rectal cancer patients. (a) Overall survival and (b) disease-free survival according to NLN in all patients.

This study included 311 consecutive patients with pathologically confirmed T3N0 rectal cancer who underwent curative surgical resection at the Sixth Affiliated Hospital of Sun Yat-sen University. Of the total cohort (Table 1), 113 patients (36.3%) were under 60 years old, while 198 patients (63.7%) were 60 years or older. Male patients comprised 210 cases (67.5%), and female patients accounted for 101 cases (32.5%). Nerve infiltration was detected in 34 patients (10.9%) and vascular invasion was observed in 28 patients (9%). Using ROC curves (Figure 1), we identified an optimal cutoff value of 21 for the number of NLNs, with patients categorized as having high NLNs (≥ 21) or low NLNs (< 21). One hundred and forty one patients (45.3%) were assigned to the high NLN group, and 170 patients (54.7%) to the low NLNs group. AC was administered to 23.4% of patients in the high NLNs group and 28.2% of patients in the low NLN group. The baseline characteristics between the two groups were generally balanced (Table 1). Consistent with previous reports [23], patients with high NLNs had significantly better survival outcomes. The high NLN group had a significantly higher 5-year OS (99.3% vs. 88.2%, *p* < 0.05) and 5-year DFS rate (92.2% vs. 79.4%, *p* < 0.05) compared to those with NLNs < 21 (Figures 2(a) and 2(b)).

### 3.2. Association of NLNs With AC in T3N0 Rectal Cancer

**Figure 3:**
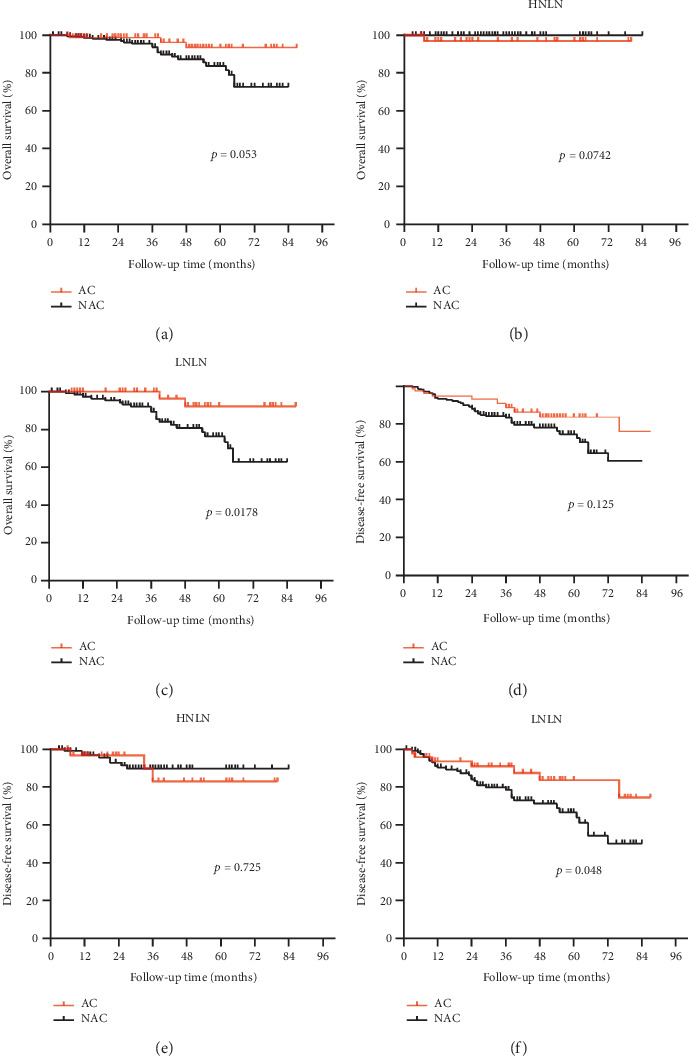
Survival outcomes according to AC and subgroup analysis. (a) Overall survival and (d) disease-free survival according to AC or NAC. (b) Overall survival and (e) disease-free survival according to AC or NAC in the HNLN group. (c) Overall survival and (f) disease-free survival according to AC or NAC in the LNLN group.

We conducted further analyses to investigate the association between the NLNs and the administration of AC in T3N0 rectal cancer. Of the 311 patients in our study, 141 (45.3%) were categorized into the high NLN group (NLNs ≥ 21) and 170 (54.7%) into the low NLN group (NLNs < 21). AC was administered to 23.4% of patients in the high NLN group and 28.2% of patients in the low NLN group. Overall, AC did not provide a significant benefit in patient prognosis compared to those who did not receive it (Figures 3(a) and 3(d)). However, in the low NLN group, we observed improved survival in patients who received AC compared to those who did not. Specifically, patient OS was significantly higher in those who received AC (95.8% vs. 82.0%, *p* < 0.05), as was DFS (85.4% vs. 72.1%, *p* < 0.05) (Figures 3(c) and 3(f)). In contrast, AC did not significantly affect OS (97.0% vs. 100%, *p* = 0.07) or DFS (90.9% vs. 92.6%, *p* = 0.73) in the high NLN group (Figures 3(b) and 3(e)). Therefore, our results suggest that T3N0 rectal cancer patients with low NLNs may benefit from AC.

### 3.3. Cox Proportional Hazards Model Predicts Risk Factors for T3N0 Rectal Cancer

Table 2 summarizes the results of both univariable and multivariable Cox proportional hazards models for OS. Both univariate and multivariate Cox regression analyses revealed that high NLNs were independent prognostic indicators of OS (hazard ratio (HR) = 0.078, 95% confidence interval (CI): 0.011–0.582, *p* = 0.013). Similarly, Table 3 presents the results of both univariable and multivariable Cox proportional hazards models for DFS. Univariable analysis identified nerve infiltration and the number of NLNs as significant factors influencing DFS (*p* < 0.05). In the multivariable analysis, nerve infiltration emerged as an independent risk factor for DFS (HR = 2.3, 95% CI: 1.117–4.737, *p* = 0.024), while a higher count of NLNs was confirmed as an independent protective factor (HR = 0.417, 95% CI: 0.213–0.815, *p* = 0.011).

## 4. Discussion

Colorectal cancer is a major contributor to newly diagnosed cancers and related deaths worldwide, accounting for nearly 10% of cases [24]. Despite advances in treatment, accurately predicting its prognosis remains a challenge. Previous studies have identified NLNs as a prognostic factor in various cancers, including breast [25], gastric [26], and colorectal [27]. In our study, we observed that patients with less than 21 NLNs identified in surgical specimens had poorer OS and DFS compared to those with 21 or more NLNs evaluated. Moreover, our Cox regression analysis demonstrated that the number of NLNs was an independent protective factor, with patient prognosis significantly improving as the number of NLNs increased.

The optimal cutoff value for the number of NLNs remains a topic of debate, with varying stratification methods employed across different studies [28, 29]. In our study, we employed ROC curve analysis and identified a cutoff value of ≥ 21 for the number of NLNs. However, Sun et al. [22] reported a cutoff value of ≥ 17 for locally advanced rectal cancer after neoadjuvant chemoradiotherapy. These discrepancies may be due to differences in patient characteristics or treatment approaches. Further research is required to establish a consensus on the optimal cutoff value for NLNs. While the prognostic significance of NLNs in cancer is increasingly acknowledged, the underlying mechanisms that explain the correlation between increased NLNs and improved survival remain unknown. Some studies have suggested that the correlation may be due to accurate tumor staging, effective surgical intervention, and high-quality pathology services. Others have implicated the immune response of the tumor and the host. It is possible that patients with higher NLNs may exhibit stronger anticancer immune responses to the tumor, leading to better survival outcomes [30].

Postoperative adjuvant therapy for Stage II colorectal cancer remains a topic of ongoing debate [31]. While some studies have demonstrated that AC can improve survival [32, 33], several others have indicated that there is no significant difference in survival between patients who received AC and those who did not [34]. In our study, multivariate Cox analysis showed no significant difference in OS and DFS between patients who received AC and those who did not in T3N0 stage rectal cancer. However, when we performed a subgroup analysis for NLNs, we found that AC did not significantly improve survival in the high NLN group. In contrast, postoperative AC was associated with improved prognosis in the low NLN group. These findings suggest that postoperative adjuvant therapy should be stratified by the number of NLNs in patients with T3N0 stage rectal cancer. It is important to note that AC can have both positive and negative effects, as it not only targets cancer cells but also healthy cells, and can cause various toxic side effects such as nausea, vomiting, and peripheral neuropathy [35, 36]. Moreover, oxaliplatin can cause significant morbidity [37]. To minimize the adverse effects of chemotherapy, the ESMO (European Society for Medical Oncology) guidelines [38] recommend follow-up observation rather than chemotherapy for Stage IIA colon cancer with no risk factors. For patients with Stage II colon cancer who are over 70 years of age, chemotherapy is recommended by Sakin et al. [39]. The NCCN guidelines recommend surgery alone as the appropriate treatment option for adequately staged, low-risk, upper rectal T3N0 tumors. However, it is worth noting that many physicians tend to ignore the NLNs when deciding on chemotherapy for patients with T3N0 rectal cancer. Our study suggests that patients with high NLNs may not require postoperative AC and can achieve a good prognosis in T3N0 rectal cancer. This can help physicians make more informed chemotherapy decisions for this patient population.

While this study provides significant findings, it is important to acknowledge several limitations that should be considered. Firstly, this study is retrospective and was conducted at a single center, which may introduce selection bias and other confounding factors. Therefore, future studies including multicenter data or prospective studies are needed to validate the conclusions reached in this study. Secondly, it is possible that differences in surgical procedures and individual surgeons may have influenced the number of LNs examined and, subsequently, our results. Future investigations should address these limitations to improve the reliability and generalizability of our findings.

## 5. Conclusion

The results of this study suggest that the number of NLNs is an independent prognostic factor for patients with T3N0 rectal cancer, with an improved prognosis observed as the number of NLNs increases. Specifically, our findings suggest that patients with a low number of NLNs (NLN < 21) may benefit from AC.

## Figures and Tables

**Table 1:** The baseline characteristics of negative lymph nodes.

**Characteristics**	**Total (** **n**%**)**	**LNLN (** **n** = 170**)**	**HNLN (** **n** = 141**)**	**p**
Age, *n*(%)				
< 60	113 (36.33)	56 (32.94)	57 (40.43)	0.172
≥ 60	198 (63.67)	114 (67.06)	84 (59.57)	
Sex, *n*(%)				
Female	101 (32.48)	57 (33.53)	44 (31.21)	0.663
Male	210 (67.52)	113 (66.47)	97 (68.79)	
Nerve invasion, *n*(%)				
No	277 (89.07)	155 (91.18)	122 (86.52)	0.191
Yes	34 (10.93)	15 (8.82)	19 (13.48)	
Vascular invasion, *n*(%)				
No	283 (91.00)	150 (88.24)	133 (94.33)	0.062
Yes	28 (9.00)	20 (11.76)	8 (5.67)	
Histologic type, *n*(%)				
Adenocarcinoma	302 (97.11)	164 (96.47)	138 (97.87)	0.463
Mucinous adenocarcinoma	9 (2.89)	6 (3.53)	3 (2.13)	
Adjuvant chemotherapy, *n*(%)				
No	230 (73.95)	122 (71.76)	108 (76.60)	0.334
Yes	81 (26.05)	48 (28.24)	33 (23.40)	

Abbreviations: HNLN, high negative lymph node; LNLN, low negative lymph node.

**Table 2:** Univariate and multivariate survival analyses of T3N0 rectal cancer (OS).

	**Univariable HR (95% CI)**	**p** ** value**	**Multivariable HR (95% CI)**	**p** ** value**
Sex		0.67		
Female	1			
Male	1.209 (0.506–2.896)			
Age		0.057		
< 60	1			
≥ 60	2.591 (0.971–6.909)			
Histologic type, *n*(%)		0.537		
Adenocarcinoma	1			
Mucinous adenocarcinoma	1.881 (0.253–13.976)			
Adjuvant chemotherapy		0.067		
No	1			
Yes	0.324 (0.097–1.083)			
NLN		0.013∗		0.013∗
LNLN	1		1	
HNLN	0.078 (0.011–0.582)		0.078 (0.011–0.582)	
Nerve invasion		0.077∗		
No	1			
Yes	2.652 (0.9–7.815)			
Vascular cancer thrombus		0.814		
No	1			
Yes	1.19 (0.28–5.058)			

Abbreviations: HNLN, high negative lymph node; LNLN, low negative lymph node; NLN, negative lymph node.

∗*p* < 0.05.

**Table 3:** Univariate and multivariate survival analyses of T3N0 rectal cancer (DFS).

	**Univariable HR (95% CI)**	**p** ** value**	**Multivariable HR (95% CI)**	**p** ** value**
Sex		0.125		
Female	1			
Male	1.67 (0.869–3.161)			
Age		0.063		
< 60	1			
≥ 60	1.797 (0.970–3.314)			
Histologic type, *n*(%)		0.871		
Adenocarcinoma	1			
Mucinous adenocarcinoma	0.849 (0.117–6.159)			
Adjuvant chemotherapy		0.131		
No	0.586			
Yes	0.586 (0.293–1.171)			
NLN		0.01∗		0.011∗
LNLN	1		1	
HNLN	0.416 (0.213–0.812)		0.417 (0.213–0.815)	
Nerve invasion		0.023∗		0.024∗
No	1		1	
Yes	2.322 (1.126–4.788)		2.3 (1.117–4.737)	
Vascular cancer thrombus		0.124		
No	1			
Yes	1.87 (0.842–4.156)			

Abbreviations: HNLN, high negative lymph node; LNLN, low negative lymph node; NLN, negative lymph node.

∗*p* < 0.05.

## Data Availability

The data of this study are available from the corresponding author upon reasonable request.
